# Long-term ischaemic and bleeding outcomes after primary percutaneous coronary intervention for ST-elevation myocardial infarction in the elderly

**DOI:** 10.1007/s12471-015-0733-2

**Published:** 2015-08-11

**Authors:** Bimmer E.P.M. Claessen, Wouter J. Kikkert, Loes P. Hoebers, Hassina Bahadurzada, Marije M. Vis, Jan Baan, Karel T. Koch, Robbert J. de Winter, Jan G.P. Tijssen, Jan J. Piek, José P.S. Henriques

**Affiliations:** Department of Cardiology, B2-115, Academic Medical Center – University of Amsterdam, Meibergdreef 9, 1105 Amsterdam, AZ The Netherlands

**Keywords:** Primary percutaneous coronary intervention, Acute myocardial infarction, Stent, Elderly

## Abstract

**Background:**

The population is ageing rapidly and the proportion of patients aged ≥ 80 years undergoing primary percutaneous coronary intervention (PCI) is rising, but clinical trials have primarily been performed in younger patients.

**Methods:**

Patients undergoing primary PCI between 2003 and 2008 were subdivided into 3 groups: < 60, 60-79, and ≥ 80 years. Endpoints at 3-year follow-up included all-cause mortality, recurrent myocardial infarction (reMI), stent thrombosis, target lesion revascularisation (TLR), bleeding (BARC bleeding ≥ 3), stroke, and major adverse cardiovascular events (MACE, a composite of cardiac mortality, reMI, stroke and TLR).

**Results:**

2002 patients with ST-segment elevation myocardial infarction (STEMI) were included, 885 (44.2 %) aged < 60, 921 (46.0 %) 60–79, and 196 (9.7 %) ≥ 80 years. Comorbidities such as diabetes mellitus, prior stroke, malignant disease, anaemia, and chronic kidney disease were more prevalent in patients ≥ 80 years. The incidence of both ischaemic and bleeding events strongly increased with age. Age ≥ 80 years was an independent predictor of mortality (HR 2.56, 95 % CI1.69–3.87, *p* < 0.001), a borderline non-significant predictor of overall bleeding (HR 1.38, 95 %CI 0.95–2.00, *p* = 0.088), and a significant predictor of non-access site bleeding (HR 2.26, 95 %CI 1.46–3.51, *p* < 0.001).

**Conclusion:**

Patients ≥ 80 years experienced high rates of ischaemic and bleeding complications; especially in this high-risk patient group individualised therapy is needed to optimise clinical outcomes.

**Electronic Supplementary Material:**

The online version of this article (doi:10.1007/s12471-015-0733-2 contains supplementary material, which is available to authorized users.

## Introduction

For patients with ST-segment elevation myocardial infarction (STEMI), primary percutaneous coronary intervention (PCI) is the preferred therapy [1, 2]. Guidelines have been composed to suggest optimal antithrombotic and antiplatelet regimens in order to prevent new ischaemic events while balancing the risk of bleeding events [2, 3]. However, due to the frailty that comes with advanced age, the higher prevalence of diverse comorbidities in the elderly, and the fact that large clinical trials often list age ≥ 80years as an exclusion criterion, it is currently unclear if following guideline recommendations will also result in acceptable outcomes regarding ischaemic and bleeding endpoints in elderly patients undergoing primary PCI.

The mean age of the population in the developed world is increasing rapidly [4]. In the European Union, the proportion of people aged ≥ 80years is expected to triple from 4.8 to 12.0 % between 2010 and 2060 [5]. An increase in the absolute and relative number of octogenarians undergoing primary PCI has been described [6]. A small number of observational studies have reported mortality rates after primary PCI for STEMI in patients aged ≥ 80 years [6–9]. However, there is a paucity of data on the long-term occurrence of recurrent ischaemic events and bleeding in the elderly after primary PCI.

We therefore investigated a cohort of STEMI patients undergoing primary PCI in a large tertiary care centre to provide more insight into ischaemic and bleeding outcomes after primary PCI for STEMI in patients aged 80 years and older.

## Methods

Data analysed in this study were obtained from STEMI patients who were accepted for primary PCI at the Academic Medical Center–University of Amsterdam between 1 January 2003, and 31 July 2008. The study complied with the Declaration of Helsinki, and the local ethics committee approved the study protocol. In general, patients qualified for primary PCI if they had typical ischaemic chest pain and at least 1 mm ST-segment elevation in two or more contiguous leads or a new left bundle branch block (LBBB). The primary PCI and adjunctive pharmacological treatment were performed according to the American College of Cardiology/American Heart Association and the European Society of Cardiology (ESC) guidelines. Patients received a standard 300–600 mg clopidogrel loading dose. If a coronary stent was implanted, clopidogrel was prescribed for at least one month to patients with a bare metal stent (BMS) and for 6 to 12 months to patients with a drug-eluting stent (DES).Patients were routinely pretreated with 300–500 mg aspirin and 5000 IU unfractionated heparin (UFH). Glycoprotein IIb/IIIa inhibitors (GPIs) were used in a bail-out setting at the discretion of the operator.

A detailed description of the study protocol has been previously published [10–12]. In short, procedural and angiographic data were prospectively collected by interventional cardiologists and specialised nurses in a dedicated database. We obtained follow-up of clinical outcome, including recurrent MI, stroke, stent thrombosis and bleeding, by reviewing inpatient and outpatient charts in the tertiary PCI centre and referring hospitals between 2011 and 2012 for consecutive STEMI patients with available activated partial thromboplastin time (aPTT) measurements in the context of a study designed to investigate the relationship between aPTT and clinical outcome in STEMI patients treated with primary PCI. Follow-up of clinical events was censored at the date of chart review. Patients whose whereabouts were unknown were considered lost to follow-up from the date of last known medical contact. Follow-up information regarding vital status was obtained from computerised, long-term mortality records from the National Death Index between 1 December 2012 and 30 April 2012.

### Study design

The study cohort consisted of all STEMI patients included in our study database who were alive at the end of the procedure. Clinical outcomes included major adverse cardiac events (MACE; a composite of cardiac death, recurrent myocardial infarction (MI), stroke and target lesion revascularisation (TLR)), all-cause, cardiac and non-cardiac mortality, recurrent MI, ischaemic and haemorrhagic stroke, stent thrombosis, TLR and bleeding. Cardiac death, recurrent MI, stent thrombosis and TLR were defined according to the Academic Research Consortium (ARC) criteria [13]. Bleeding complications were defined according to the Bleeding Academic Research Consortium (BARC) bleeding classification and the Thrombolysis In Myocardial Infarction bleeding classification [14, 15] Stroke was defined as an irreversible neurological deficit, as classified by the treating neurologist, on the basis of supporting information, including brain images and neurological evaluation.

### Statistical analysis

Normally distributed continuous variables are reported as the mean with standard deviation and compared with the one-way ANOVA test, skewed distributed variables are presented as the median with interquartile range (IQR) and compared with the Kruskal-Wallis test. Categorical variables are presented as proportions and compared with the χ^2^ test for trend. Event rates of clinical outcomes were estimated using Kaplan-Meier analyses. Missing covariate data were imputed with the use of multiple imputations and were assumed to be missing at random. We performed 10 imputations using the Markov Chain Monte Carlo method. Cox proportional hazards models were first fit separately in the 10 imputed datasets and subsequently pooled according to Rubin’s protocol [16]. Multivariable Cox proportional hazards regression was used in the following manner: first, univariable hazard ratios were calculated for all variables in Tables 1 and 2. Then, multivariable backward stepwise Cox regression models were constructed using variables with *p* < 0.10 in univariate analysis as candidate variables. Entry and exit criteria were set at *p* = 0.10.

In order to plot the graphic relationships between age and probability of death, MACE, BARC type ≥ 3 bleeding and stroke at 3 years, we calculated the predicted probability of these events by incorporating the coefficients returned by the unadjusted and adjusted Cox models in the following equation:

$$S\left( t \right)={{e}^{\left( -{{H}_{o}}\left( t \right)*\left({{x}_{1}}{{\beta }_{1}}+{{x}_{2}}{{\beta }_{2}}+\ldots{{x}_{k}}{{\beta }_{k}} \right) \right)}}$$

Where S(t) is the predicted event-free survival at timepoint t, H_0_(t) is the baseline hazard at time t, x_1, 2, … k_ is the vector of the covariates, and β_1, 2, … k_ the coefficients determined from the Cox regression models. The risk of adverse events was calculated as 1 min S(t). For the present analysis, we assumed ‘t’ to be 1095 days for all patients. The predicted probabilities were pooled over the imputed datasets utilising Rubin’s protocol after applying the logit transformation to the predicted probabilities [17]. The relationship between age and the probability of death, MACE, stroke and bleeding were plotted graphically using a model fitting approach involving cubic polynomials (splines) as previously employed by the GUSTO-I (Global Utilization of Streptokinase and Tissue Plasminogen Activator for Occluded Coronary Arteries) trial investigators [18]. All analyses were performed with Statistical Package for Social Sciences software (SPSS version 19.0, Chicago, Illinois).

## Results

Of 2009 STEMI patients recorded in our database who were treated with primary PCI in our institution between 1 January 2003 and 31 July 2008, 2002 were alive at the end of the procedure. Median follow-up duration in this cohort of patients was 4.9 years (IQR 3.4–6.4 years). A total of 196 patients were aged 80 years or older (9.7 %), 921 patients were aged 60–79 years (46.0 %), and 885 patients were aged < 60 years (44.2 %).

Baseline characteristics are shown in Table 1. The proportion of female patients increased with age; among patients aged ≥ 80 years the majority of patients were female. Patients aged ≥ 80 years were more likely to have diabetes mellitus, prior coronary artery bypass graft surgery (CABG), a history of stroke or transient ischaemic event, peripheral artery disease and malignant disease compared with younger patients. Moreover, they more often had anaemia and an estimated creatinine clearance of < 60 ml/min/1.73 m^2^. On the other hand, patients aged ≥ 80 years were less likely to be smokers, less often had a positive family history of coronary artery disease and had lower rates of hypercholesterolaemia at admission compared with younger patients.

Angiographic and procedural characteristics are shown in Table 2. Patients aged ≥ 80 years had longer ischaemic times, a higher prevalence of cardiogenic shock at admission, lower rates of post-procedural TIMI 3 flow, a higher rate of coronary calcification, multivessel disease and chronic total occlusions in non-infarct related vessels compared with younger patients.

**Fig. 1:**
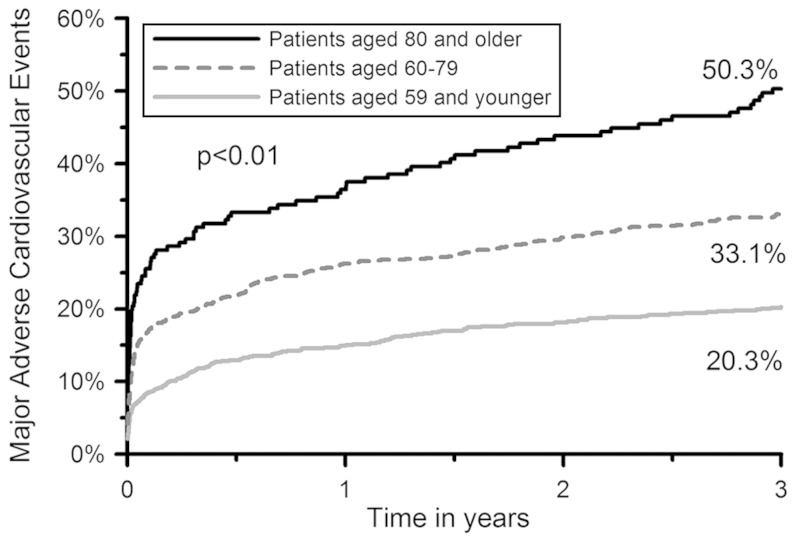
Kaplan-Meier curve for major adverse cardiac events

**Fig. 2:**
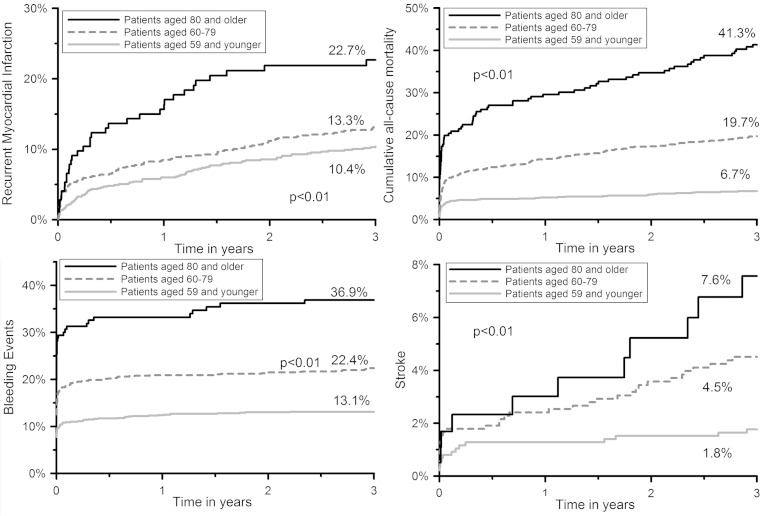
Kaplan-Meier curve for recurrent myocardial infarction, mortality, BARC bleeding ≥ 3, and stroke. *BARC* bleeding academic research consortium

Figure 1 displays a Kaplan-Meier time-to-event curve for MACE. Figure 2 displays Kaplan-Meier time-to-event curves for recurrent MI, mortality, BARC bleeding ≥ 3, and stroke. Table 3 shows Kaplan-Meier estimates of the incidence of adverse events at 3-year follow-up. Rates of MACE, mortality, recurrent MI, stroke and BARC bleeding ≥ 3 increased with age. Conversely, rates of stent thrombosis and target lesion revascularisation decreased non-significantly with age.

Table 1 of the Supplementary Appendix shows the results of multivariable Cox regression analyses for 3-year clinical outcomes. Relative to patients aged < 60 years, patients aged 60–79 years and those aged ≥ 80 years were found to have an increased risk of cardiac and non-cardiac mortality, stroke and non-access site bleeding after correction for confounding variables. In addition, age 60–79 years and age ≥ 80 years was associated with a borderline non-significant increase in risk of BARC type ≥ 3 bleeding. On the other hand, age ≥ 80 years was not an independent predictor of MACE. Detailed multivariable models are shown in the Supplementary Appendix.

**Fig. 3:**
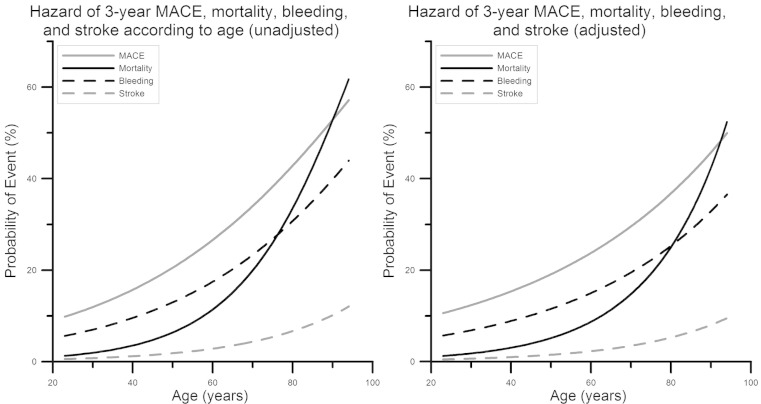
Probability of 3-year mortality, major adverse cardiac events, stroke, and BARC bleeding ≥ 3 according to age. *MACE* major adverse cardiovascular event, *BARC* bleeding academic research consortium

Age as a continuous variable was an independent predictor of all-cause mortality (1.43 per 10 year increase; 95 % CI 1.27–1.61; *p* < 0.001), cardiac mortality (HR 1.34; 95 % CI 1.17–1.53, *p* < 0.001), non-cardiac mortality (HR 1.55; 95 % CI 1.24–1.95, *p* < 0.001), stroke (HR 1.46; 95 % CI 1.18–1.80; *p* < 0.001) and BARC type ≥ 3 bleeding (HR 1.15; 95 % CI 1.04–1.26; *p* = 0.006), but not of MACE (HR 1.06; 95 % CI 0.98–1.16; *p* = 0.17), or recurrent MI (HR 1.04; 95 % CI 0.93–1.17, *p* = 0.49) (Supplementary Appendix, Table 2). Figure 3 shows a graphic representation of the unadjusted (left panel) and adjusted (right panel) relation between age as a continuous variable and the hazard of MACE, mortality, BARC bleeding ≥ 3, and stroke up to 3-year follow-up.

## Discussion

The current study extends previous reports by providing insight into the occurrence of a large number of clinically relevant endpoints in up to 3 years of follow-up in patients undergoing primary PCI according to age. We found higher rates of ischaemic and haemorrhagic endpoints with increasing age, illustrating the frailty of this rapidly growing high-risk patient group.

In a recent large registry, Velders et al. found an increased risk of early mortality and heart failure in patients aged ≥ 80 as compared with patients younger than 80 [8]. However, among the elderly patients who survived the first month(s), the prognosis was similar compared with their younger counterparts. This indicates that the difference in mortality between patients aged ≥ 80 and their younger counterparts can be explained by the direct adverse consequence of the STEMI itself, such as arrhythmia, heart failure, mechanical complications and procedural complications such as bleeding. Interestingly, consistent with previous studies, we found that elderly patients were less likely to develop stent thrombosis or to undergo target lesion revascularisation [19, 20]. The fact that the rate of recurrent MI was higher among elderly patients suggests that recurrent MI in elderly patients is the result of plaque rupture of non-target lesions, and is likely explained by the more extensive atherosclerosis among the elderly.

The incidence of both access site and non-access site bleeding strongly increased with age in this study. Well-known risk factors for bleeding such as female gender and chronic kidney disease were more prevalent in the elderly [21]. Moreover, advanced age is associated with increased vascular fragility and calcification, which are known risk factors for access site bleeding [22, 23]. In the current study, age ≥ 80 years was independently associated with non-access site bleeding, an important finding given the fact that especially non-access site bleeding is associated with adverse outcome [21, 24]. In a pooled patient-level analysis involving 14,180 patients recruited in 7 randomised trials PCI treatment with a GP IIb/IIIa inhibitor was associated with a significant 27 % higher risk of non-access site bleeding [25]. A large observational study in 10,469 patients aged ≥ 80 years included in the CathPCI registry suggests that bleeding avoidance strategies such as radial access and use of vascular closure devices were independently associated with lower bleeding [26]. A number of recommendations to reduce bleeding complications in the elderly may be hypothesised: (1) the radial approach would seem to be the preferred method of vascular access in patients aged ≥ 80 years; (2) Routine proton pump inhibition might reduce gastrointestinal bleeding in patients aged ≥ 80 years; and (3) use of GP IIb/IIIa inhibitors in the elderly may lead to increased bleeding and should be avoided where possible.

By observing the Kaplan-Meier curves for bleeding, recurrent MI, and stroke, it appears that bleeding predominantly occurs during the first month after primary PCI, whereas the incidence of recurrent MI and stroke (of the ischaemic type in the vast majority of patients) continues to increase during the 3-year follow-up period. Studies investigating novel P2Y12 inhibitors such as prasugrel and ticagrelor have reported superior outcomes compared with clopidogrel with regards to ischaemic events, at a cost of increased bleeding events [27, 28]. No studies investigating strategies to reduce MI and stroke after primary PCI for STEMI in patients ≥ 80 years have been conducted yet. It is therefore currently still unclear whether there is a role for novel P2Y12 inhibitors in this population.

Recently, a small number of observational studies have reported results of primary PCI for STEMI in patients aged ≥ 80 years, showing 30-day mortality rates of around 28 % [6–8]. The high rates of mortality in patients aged ≥ 80 years can largely be explained by a higher burden of comorbidities, a higher rate of haemodynamic instability during primary PCI, and high rates of bleeding and recurrent MI as shown in the current study. While acknowledging that advanced age itself is an important and unmodifiable predictor of mortality, a strategy aimed at reducing early bleeding complications, and late ischaemic complications might potentially lead to improved mortality in patients aged ≥ 80 years. Recent developments in primary PCI associated with superior outcomes such as the radial rather than the femoral approach becoming the default vascular access strategy in primary PCI, and the introduction of more effective antiplatelet agents such as prasugrel and ticagrelor instead of clopidogrel may have the greatest absolute benefit in patients aged ≥ 80 years, as these patients also have the highest absolute event rates of adverse events [29]. Nonetheless, the novel P2Y12 inhibitors that are relatively more effective in inhibiting platelet aggregation compared with clopidogrel may also cause more bleeding events in the elderly, potentially offsetting the reduction in ischaemic events observed in randomised controlled trials. Ideally, this should be investigated in a randomised clinical trial enrolling STEMI patients aged ≥ 80 years.

### Limitations

A number of limitations of the present study deserve mentioning. First, only a relatively small cohort of patients aged ≥ 80 years were included in the present analysis. Nonetheless, this is the largest cohort of very elderly patients to date with detailed reporting of a multitude of 3-year ischaemic and bleeding events. Moreover, due to the high event rates, this cohort was sufficiently powered to conduct multivariable analyses. Finally, as this was an observational study we can only speculate about treatment strategies to improve the clinical outcome of very elderly patients undergoing primary PCI.

## Conclusion

Patients ≥ 80 years experienced high rates of ischaemic and bleeding complications, especially in this high-risk patient group individualised therapy is needed to optimise clinical outcomes.

### Funding source

This work was supported by The Nuts OHRA Foundation, the Netherlands [SNO-T-0702-61]

## Electronic Supplementary Material

Supplementary Material 1 (DOCX 62 KB)
