# Supplementary material for: Long-term ischaemic and bleeding outcomes after primary percutaneous coronary intervention for ST-elevation myocardial infarction in the elderly
Source: Neth Heart J. 2015 Aug 11;23(10):477–82. doi: 10.1007/s12471-015-0733-2 (PMC4580666; doi:10.1007/s12471-015-0733-2)
Supplement: Supplementary file 1 — Supplementary Material 1 (DOCX 62 KB) [file 12471_2015_733_MOESM1_ESM.docx]

**Supplementary Appendix**

B. E. Claessen*, W. J. Kikkert*, L. P. Hoebers, H. Bahadurzada, M. M. Vis, J. Baan Jr, K. T. Koch, R. J. de Winter, J. G. P. Tijssen, J. J. Piek, J. P.S. Henriques

| Table 1. Percentage of missing data for table 1. | | |
| --- | --- | --- |
|  | | % missing data |
| Male | | 0 |
| Age | | 0 |
| BMI | | 10.0 |
| History of | |  |
|  | Diabetes | 0 |
|  | Current smoking | 0 |
|  | Hypertension | 0 |
|  | Hypercholesterolaemia | 0 |
|  | Previous stroke or TIA | 0 |
|  | Peripheral vascular disease | 0 |
|  | Bleeding | 0 |
|  | Malignant disease | 0 |
|  | Recent surgery (<7 days) | 0 |
|  | Previous MI | 0 |
|  | Previous PCI | 0 |
|  | Previous CABG | 0 |
|  | Family history CAD | 0 |
| Laboratory values | |  |
| Anaemia | | 0.1 |
| White blood cell count | | 1.2 |
| Creatinine clearance | | 9.2 |
| Thrombocyte count | | 1.0 |

| Table 2. Procedural and angiographic characteristics | |
| --- | --- |
|  | % missing data |
| Total ischaemic time | 9.6 |
| Cardiogenic shock | 0.8 |
| IABP/Impella | 0.1 |
| Loading dose clopidogrel | 2.9 |
| Glycoprotein IIb/IIIa Inhibitor | 0 |
| PCI access site | 0.1 |
| Infarct related artery | 3.2 |
| Pre-procedural TIMI flow in IRA | 10.0 |
| Post-procedural TIMI flow in culprit artery | 5.5 |
| Multivessel disease | 3.8 |
| Chronic total occlusion in non-IRA | 3.8 |
| Ostial lesion | 4.1 |
| Calcification | 4.1 |
| Amount of lesions treated | 0 |
| Stent | 0 |
| Stent length – mm (median (IQR)) [available in] | 0.2 |

| Table 3. Multivariable predictors of MACE within 3 years | | | | | | |
| --- | --- | --- | --- | --- | --- | --- |
|  | |  | HR | 95% CI | | p-value |
| Peripheral artery disease | | | 1.66 | 1.28 | 2.17 | < 0.001 |
| Male gender | | | 0.80 | 0.66 | 0.96 | 0.016 |
| IABP | |  | 1.30 | 1.00 | 1.68 | 0.046 |
| Current smoker | | | 0.82 | 0.68 | 0.98 | 0.033 |
| Cardiogenic shock | | | 1.66 | 1.25 | 2.21 | 0.001 |
| Multivessel disease without concurrent CTO | | | 1.41 | 1.14 | 1.74 | 0.001 |
| Multivessel disease with concurrent CTO | | | 1.77 | 1.42 | 2.22 | < 0.001 |
| Infarct-related artery | | |  |  |  |  |
|  | RCA/LCx | | 1.00 | … | … | … |
|  | LM/LAD | | 1.23 | 1.04 | 1.47 | 0.018 |
| Total ischaemic time (per 30 min increment) | | | 1.01 | 1.00 | 1.01 | 0.028 |
| Thrombocyte count | | |  |  |  |  |
|  | < 150 | | 1.95 | 1.36 | 2.79 | < 0.001 |
|  | 150 – 400 | | 1.00 | … | … | … |
|  | > 400 | | 1.02 | 0.67 | 1.55 | 0.916 |
| Calcification IRA | | | 1.37 | 1.13 | 1.65 | 0.001 |
| TIMI flow post-procedure | | |  |  |  |  |
|  | 0/1 | | 2.27 | 1.61 | 3.18 | < 0.001 |
|  | 2/3 | | 1.00 | … | … | … |
| White blood cell count | | |  |  |  |  |
|  | < 11 | | 1.00 | … | … | … |
|  | ≥ 11 | | 1.27 | 1.07 | 1.52 | 0.007 |
| Anaemia | | | 1.57 | 1.27 | 1.93 | < 0.001 |
| Creatinine clearance | | |  |  |  |  |
|  | < 60 ml/min | | 1.59 | 1.29 | 1.96 | < 0.001 |
|  | ≥ 60 ml/min | | 1.00 | … | … | … |
| Arterial access site | | |  |  |  |  |
|  | Femoral artery | | |  |  |  |
|  | Radial artery | | 1.24 | 0.82 | 1.89 | 0.304 |
|  | Other or combinations | | 2.48 | 1.29 | 4.77 | 0.006 |
| Previous PCI | | | 1.32 | 1.02 | 1.71 | 0.034 |
| GP IIb/IIIa inhibitor | | | 1.25 | 1.04 | 1.50 | 0.017 |

*MACE* major adverse cardiac events; *HR* hazard ratio; *CI* confidence interval; *IABP* intra-aortic balloon pump; *CTO* chronic total occlusion;

| Table 4. Multivariable predictors of all-cause mortality within 3 years of follow-up | | | | | |
| --- | --- | --- | --- | --- | --- |
|  |  | HR | 95% CI | | p-value |
| PCI access site | |  |  |  |  |
|  | Femoral | 1.00 | … | … | … |
|  | Radial | 2.06 | 1.31 | 3.24 | 0.002 |
|  | Other or combinations | 3.24 | 1.59 | 6.62 | 0.001 |
| IABP |  | 1.52 | 1.08 | 2.12 | 0.016 |
| Cardiogenic shock | | 1.93 | 1.34 | 2.77 | < 0.001 |
| History of malignant disease | | 1.88 | 1.41 | 2.51 | < 0.001 |
| Family history of coronary artery disease | | 0.54 | 0.40 | 0.72 | < 0.001 |
| Thrombocyte count | |  |  |  |  |
|  | < 150 | 2.21 | 1.48 | 3.29 | < 0.001 |
|  | 150 – 400 | 1.00 | … | … | … |
|  | > 400 | 1.37 | 0.85 | 2.23 | 0.200 |
| TIMI flow post procedure | | |  |  |  |
|  | 0/1 | 1.85 | 1.23 | 2.78 | 0.003 |
|  | 2/3 | 1.00 | … | … | … |
| Infarct-related artery | | |  |  |  |
|  | RCA/LCx | 1.00 | … | … | … |
|  | LM/LAD | 1.31 | 1.04 | 1.64 | 0.024 |
| Multivessel disease without concurrent CTO | | 1.39 | 1.05 | 1.83 | 0.021 |
| Multivessel disease with concurrent CTO | | 1.64 | 1.23 | 2.19 | 0.001 |
| White blood cell count | | |  |  |  |
|  | < 11 | 1.00 | … | … | … |
|  | ≥ 11 | 1.62 | 1.28 | 2.05 | < 0.001 |
| Anaemia |  | 2.07 | 1.60 | 2.67 | < 0.001 |
| Creatinine clearance | | |  |  |  |
|  | < 60 ml/min | 1.57 | 1.18 | 2.09 | 0.002 |
|  | ≥ 60 ml/min | 1.00 | … | … | … |
| Age |  |  |  |  |  |
|  | < 60 years | 1.00 | … | … | … |
|  | 60 – 79 | 1.87 | 1.36 | 2.56 | < 0.001 |
|  | ≥ 80 years | 2.56 | 1.69 | 3.87 | < 0.001 |
| History of stroke or TIA | | 1.44 | 1.00 | 2.06 | 0.050 |
| Peripheral artery disease | | 1.39 | 1.01 | 1.92 | 0.046 |

| Table 5. Multivariable predictors of cardiac mortality within 3 years of follow-up | | | | | | |
| --- | --- | --- | --- | --- | --- | --- |
|  | |  | HR | 95% CI | | p-value |
| Multivessel disease without concurrent CTO | | | 1.83 | 1.34 | 2.51 | < 0.001 |
| Multivessel disease with concurrent CTO | | | 2.48 | 1.81 | 3.41 | < 0.001 |
| Infarct-related artery | | | |  |  |  |
|  | RCA/LCx | | 1.00 | … | … | … |
|  | LM/LAD | | 1.58 | 1.23 | 2.02 | < 0.001 |
| Thrombocyte count | | |  |  |  |  |
|  | < 150 | | 3.01 | 1.97 | 4.61 | < 0.001 |
|  | 150 – 400 | | 1.00 | … | … | … |
|  | > 400 | | 1.34 | 0.77 | 2.33 | 0.308 |
| TIMI flow post-procedure | | | |  |  |  |
|  | 0/1 | | 2.31 | 1.49 | 3.58 | < 0.001 |
|  | 2/3 | | 1.00 | … | … | … |
| White blood cell count | | | |  |  |  |
|  | < 11 | | 1.00 | … | … | … |
|  | ≥ 11 | | 1.96 | 1.50 | 2.56 | < 0.001 |
| Anaemia | | | 2.30 | 1.73 | 3.05 | < 0.001 |
| Creatinine clearance | | | |  |  |  |
|  | < 60 ml/min | | 2.47 | 1.78 | 3.42 | < 0.001 |
|  | ≥ 60 ml/min | | 1.00 | … | … | … |
| Age | | |  |  |  |  |
|  | < 60 years | | 1.00 | … | … | … |
|  | 60 – 79 | | 1.68 | 1.18 | 2.40 | 0.004 |
|  | ≥ 80 years | | 2.34 | 1.49 | 3.69 | < 0.001 |

| Table 6. Multivariable predictors of non-cardiac mortality within 3 years of follow-up | | | | | | |
| --- | --- | --- | --- | --- | --- | --- |
|  | |  | HR | 95% CI | | p-value |
| Arterial access site | | |  |  |  |  |
|  | Femoral | | 1.00 | … | … | … |
|  | Radial | | 2.60 | 1.22 | 5.53 | 0.013 |
| History of stroke or TIA | |  | 2.24 | 1.13 | 4.45 | 0.022 |
| History of malignant disease | | | 4.06 | 2.41 | 6.83 | < 0.001 |
| GP IIb/IIIa inhibitor | | | 0.42 | 0.20 | 0.88 | 0.021 |
| Family history of CAD | | | 0.56 | 0.30 | 1.01 | 0.056 |
| Anaemia | |  | 2.61 | 1.57 | 4.33 | < 0.001 |
| Age | |  |  |  |  |  |
|  | < 60 years | | 1.00 | … | … | … |
|  | 60 – 79 | | 3.23 | 1.55 | 6.77 | 0.002 |
|  | ≥ 80 years | | 3.74 | 1.54 | 9.10 | 0.004 |
| BMI | |  |  |  |  |  |
|  | < 18.5 | | 4.21 | 1.31 | 13.6 | 0.016 |
|  | 18.5 - 29.9 | | 1.00 | … | … | … |
|  | ≥ 30 | | 1.60 | 0.87 | 2.96 | 0.131 |

| Table 7. Multivariable predictors of recurrent MI within 3 years of follow-up | | | | | |
| --- | --- | --- | --- | --- | --- |
|  |  | HR | 95% CI | | p-value |
| Peripheral artery disease | | 2.00 | 1.34 | 2.99 | 0.001 |
| Diabetes |  | 1.63 | 1.19 | 2.23 | 0.003 |
| GP IIb/IIIa inhibitor | | 1.56 | 1.19 | 2.05 | 0.001 |
| Multivessel disease without concurrent CTO | | 1.48 | 1.06 | 2.05 | 0.020 |
| Multivessel disease with concurrent CTO | | 1.84 | 1.28 | 2.66 | 0.001 |
| Prior MI |  | 1.50 | 1.07 | 2.10 | 0.019 |
| Calcification IRA | | 1.60 | 1.19 | 2.13 | 0.002 |
| Ostial lesion IRA | | 1.38 | 0.96 | 1.97 | 0.081 |
| Anaemia |  | 1.59 | 1.15 | 2.20 | 0.005 |

| Table 8. Multivariable predictors of stroke within 3 years of follow-up | | | | | | |
| --- | --- | --- | --- | --- | --- | --- |
|  | |  | HR | 95% CI | | p-value |
| History of stroke or TIA | | | 2.29 | 1.11 | 4.70 | 0.025 |
| Cardiogenic shock | | | 3.52 | 1.86 | 6.64 | 0.000 |
| TIMI flow post-procedure | | |  |  |  |  |
|  | 0/1 | | 3.30 | 1.42 | 7.68 | 0.006 |
|  | 2/3 | | 1.00 | … | … | … |
| Age | |  |  |  |  |  |
|  | < 60 years | | 1.00 | … | … | … |
|  | 60 - 79 | | 2.20 | 1.20 | 4.03 | 0.011 |
|  | ≥ 80 years | | 3.19 | 1.43 | 7.10 | 0.004 |

| Table 9. Multivariable predictors of stent thrombosis within 3 years of follow-up | | | |  |
| --- | --- | --- | --- | --- |
|  | HR | 95% CI | | p-value |
| Peripheral artery disease | 2.03 | 0.97 | 4.23 | 0.060 |
| GP IIb/IIIa inhibitor | 1.55 | 0.95 | 2.51 | 0.078 |
| Pre-existing dissection | 1.77 | 1.04 | 3.01 | 0.036 |
| Stent length | 1.02 | 1.00 | 1.03 | 0.041 |

| Table 10. Multivariable predictors of target lesion revascularisation within 3 years of follow-up | | | | | |
| --- | --- | --- | --- | --- | --- |
|  |  | HR | 95% CI |  | p-value |
| Infarct-related artery | |  |  |  |  |
|  | RCA/LCx |  |  |  |  |
|  | LM/LAD | 1.39 | 1.03 | 1.88 | 0.032 |
| Total ischaemic time (per 30 min increment) | | 1.01 | 1.01 | 1.02 | < 0.001 |
| Age |  |  |  |  |  |
|  | < 60 years | |  |  |  |
|  | 60 - 79 | 0.94 | 0.69 | 1.28 | 0.690 |
|  | ≥ 80 years | 0.32 | 0.14 | 0.69 | 0.004 |
| Peripheral artery disease | | 1.74 | 1.06 | 2.84 | 0.028 |
| Previous PCI | | 1.83 | 1.23 | 2.73 | 0.003 |
| History of hypertension | | 1.34 | 1.00 | 1.79 | 0.053 |
| IABP |  | 1.49 | 0.97 | 2.27 | 0.066 |
| Calcification of the IRA | | 1.88 | 1.34 | 2.64 | < 0.001 |
| Stenting |  | 0.64 | 0.43 | 0.96 | 0.030 |
| Number of lesions treated | | |  |  |  |
|  | 0 |  |  |  |  |
|  | 1 | 4.95 | 1.30 | 18.81 | 0.019 |
|  | 2 | 7.05 | 1.44 | 34.60 | 0.016 |

| Table 11. Multivariable predictors of BARC type ≥ 3 bleeding within 3 years follow up | | | | | |
| --- | --- | --- | --- | --- | --- |
|  |  | HR | 95% CI | | p-value |
| History of stroke or TIA |  | 1.52 | 1.08 | 2.15 | 0.018 |
| Male gender | | 0.54 | 0.44 | 0.67 | < 0.001 |
| IABP |  | 2.20 | 1.71 | 2.82 | < 0.001 |
| History of malignant disease | | 1.43 | 1.05 | 1.94 | 0.023 |
| GP IIb/IIIa inhibitor | | 1.76 | 1.41 | 2.20 | < 0.001 |
| Infarct-related artery | | |  |  |  |
|  | RCA/LCx | 1.00 | … | … | … |
|  | LM/LAD | 1.19 | 0.96 | 1.48 | 0.121 |
| Number of lesions treated | | |  |  |  |
|  | 0 | 1.00 | … | … | … |
|  | 1 | 0.56 | 0.35 | 0.90 | 0.017 |
|  | 2 | 1.02 | 0.51 | 2.01 | 0.965 |
|  | 3 | 0.30 | 0.04 | 2.32 | 0.248 |
| Stenting |  | 0.66 | 0.49 | 0.88 | 0.005 |
| Anaemia |  | 1.41 | 1.10 | 1.81 | 0.007 |
| Creatinine clearance | | |  |  |  |
|  | < 60 ml/min | 1.66 | 1.26 | 2.18 | < 0.001 |
|  | ≥ 60 ml/min | 1.00 | … | …. | …. |
|  |  |  |  |  |  |
| BMI |  |  |  |  |  |
|  | < 18.5 | 1.74 | 0.83 | 3.68 | 0.143 |
|  | 18.5 - 29.9 | 1.00 | … | … | … |
|  | ≥ 30 | 0.75 | 0.55 | 1.03 | 0.072 |
| Recent surgery | | 1.70 | 0.90 | 3.22 | 0.100 |
| Age |  |  |  |  |  |
|  | < 60 years | 1.00 | … | … | … |
|  | 60 - 79 | 1.31 | 1.03 | 1.68 | 0.030 |
|  | ≥ 80 years | 1.38 | 0.95 | 2.00 | 0.088 |

| Table 12. Multivariable predictors of access site bleeding within 3 years of follow up | | | | | |
| --- | --- | --- | --- | --- | --- |
|  |  | HR | 95% CI | | p-value |
| BMI | |  |  |  |  |
|  | < 18.5 | 1.88 | 0.76 | 4.64 | 0.168 |
|  | 18.5 – 30.0 | 1.00 | … | … | … |
|  | ≥ 30 | 0.46 | 0.29 | 0.74 | 0.002 |
| Creatinine clearance | | |  |  |  |
|  | < 60 ml/min | 1.62 | 1.13 | 2.31 | 0.008 |
|  | ≥ 60 ml/min | 1.00 | … | … | … |
| Multivessel disease without concurrent CTO | | 1.40 | 1.02 | 1.93 | 0.037 |
| Multivessel disease with concurrent CTO | | 1.30 | 0.90 | 1.88 | 0.168 |
| Stenting | | 0.70 | 0.50 | 0.98 | 0.038 |
| GP IIb/IIIa inhibitor | | 1.99 | 1.51 | 2.63 | < 0.001 |
| IABP |  | 2.25 | 1.66 | 3.05 | < 0.001 |
| Male gender | | 0.33 | 0.25 | 0.44 | < 0.001 |
| Age |  |  |  |  |  |
|  | < 60 years | |  |  |  |
|  | 60 - 79 | 1.07 | 0.77 | 1.49 | 0.678 |
|  | ≥ 80 years | 1.17 | 0.72 | 1.91 | 0.520 |
| Peripheral artery disease | | 1.55 | 1.02 | 2.36 | 0.040 |

| Table 13. Multivariable predictors of non-access site bleeding within 3 years of follow up | | | | | | |
| --- | --- | --- | --- | --- | --- | --- |
|  | |  | HR | 95% CI | | p-value |
| IABP | |  | 2.20 | 1.59 | 3.05 | < 0.001 |
| History of malignant disease | | | 1.62 | 1.09 | 2.40 | 0.017 |
| History of bleeding | | | 1.77 | 1.09 | 2.88 | 0.021 |
| GP IIb/IIIa inhibitor | | | 1.54 | 1.14 | 2.09 | 0.006 |
| Family history of coronary artery disease | | | 0.65 | 0.47 | 0.90 | 0.008 |
| Recent surgery | | | 2.17 | 1.06 | 4.42 | 0.033 |
| Number of lesions treated | | | |  |  |  |
|  | 0 | |  |  |  |  |
|  | 1 | | 0.44 | 0.23 | 0.86 | 0.016 |
|  | 2 | | 0.54 | 0.20 | 1.44 | 0.215 |
|  | 3 | | 0.39 | 0.05 | 3.22 | 0.381 |
| Thrombus IRA | | | 0.73 | 0.55 | 0.97 | 0.032 |
| Stenting | |  | 0.66 | 0.45 | 0.97 | 0.036 |
| White blood cell count | | | |  |  |  |
|  | < 11 | | 1.00 | … | … | … |
|  | ≥ 11 | | 1.39 | 1.04 | 1.86 | 0.026 |
| Anaemia |  | | 2.04 | 1.47 | 2.81 | < 0.001 |
| Age |  | |  |  |  |  |
|  | < 60 years | | |  |  |  |
|  | 60 - 79 | | 1.74 | 1.26 | 2.41 | 0.001 |
|  | ≥ 80 years | | 2.24 | 1.44 | 3.48 | < 0.001 |
